# A New Quinone Based Fluorescent Probe for High Sensitive and Selective Detection of Biothiols and Its Application in Living Cell Imaging

**DOI:** 10.1155/2019/7536431

**Published:** 2019-04-09

**Authors:** Yanju Liu, Manman Li, Keith Man-Chung Wong, Yan Tong, Huaixia Yang, Jinming Kong

**Affiliations:** ^1^Pharmacy College, Henan University of Chinese Medicine, Zhengzhou 450008, China; ^2^Department of Chemistry, Southern University of Science and Technology, Shenzhen 518055, China; ^3^School of Environmental and Biological Engineering, Nanjing University of Science and Technology, Nanjing 210094, China

## Abstract

In view of the vital role of biothiols in many physiological processes, the development of simple and efficient probe for the detection of biothiols is of great medical significance. In this work, we demonstrate the use of 2,3-dichloro-5,6-dicyano-1,4-benzoquinone (DDQ), which respond rapidly to biothiols especially to glutathione, as a new fluorescent probe for the selective detection and bioimaging of biothiols. This new fluorescent probe can distinguish glutathione from cysteine and homocysteine easily under physiological concentration and detect glutathione quickly within three minutes. This probe exhibits high selectivity to biothiols and the detection limit was determined to be 3.08 × 10^−9^ M for glutathione, 8.55 × 10^−8^ M for cysteine, and 2.17 × 10^−9^ M for homocysteine, respectively. The sensing mechanism was further explored by density functional theory (DFT) and nuclear magnetic resonance (NMR) experiment; results showed that the interaction forces between the probe and biothiols were electrostatic interaction. In addition, the probe has been successfully applied to the detection of biothiols in Eca9706 cells by fluorescence confocal imaging technology.

## 1. Introduction

Small molecular biothiols, such as cysteine (Cys), glutathione (GSH), and homocysteine (Hcy), play an important role in physiological and pathological processes [1, 2]. Researches have shown that biothiols have functions of maintaining the stability of intracellular environment, enhancing immunity, promoting cellular growth, and antiaging [3, 4], which are of great significance for human health. The contents of biothiols in human bodies are closely related to specific diseases. Cys deficiency often leads to a range of diseases such as hematopoietic dysfunction, leukopenia, liver damage, edema, and so on [5, 6], while high content of cysteine will cause neurotoxicity [7]. Meanwhile, the level of homocysteine can be used to predict the prevalence rate of heart disease, apoplexy, and Alzheimer's disease as well as act as an independent indicator of heart, brain, and peripheral vascular diseases [8–11]. Glutathione is the most abundant biothiol in human being with its concentration range from 1 to 15 mM in cells [12]; it involved in many cellular processes, including cellular redox homeostasis, intracellular signal transduction, and gene regulation [13, 14]; abnormal levels of glutathione can cause cancer and accelerate aging, heart problems, and other diseases [15, 16]. Therefore, it is of great medical significance to detect biothiols.

The methods of detecting biothiols include high performance liquid chromatography (HPLC) [23, 24], ultraviolet visible spectrometry [25, 26], electrochemical method [27, 28], capillary electrophoresis [29], mass spectrometry [30], and so forth. Among them, fluorescence method has attracted much attention due to its high sensitivity and efficiency [31–38]; in particular it can be used for real-time imaging of living tissues by means of confocal detection [39–41].

**Scheme 1:**
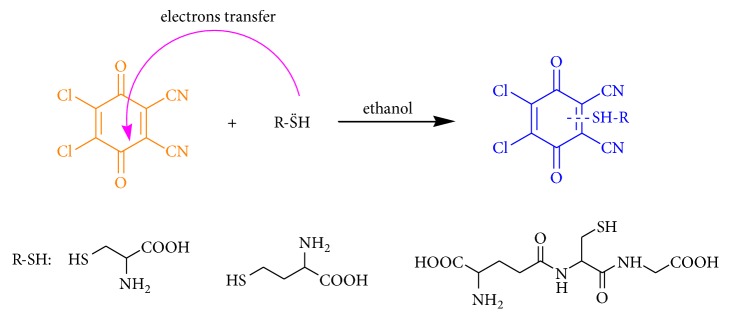
Schematic illustration of the detection for biothiols of DDQ as a probe.

2,3-Dichloro-5,6-dicyano-1,4-benzoquinone (DDQ) is one kind of* p*-benzoquinone; when reacting with biothiols, the electrons in biothiols transfer to DDQ, thus obtaining a product with high intensity fluorescence (Scheme 1). A series of experiments were designed to investigate the detection performance of DDQ; results show that the probe has high sensitivity and selectivity for the detection of biothiols and has been successfully applied to detect biothiols in Eca9706 cells by confocal imaging.

## 2. Experimental

### 2.1. Reagents and Materials

2,3-Dichloro-5,6-dicyano-1,4-benzoquinone (DDQ), cysteine (Cys), glutathione (GSH), homocysteine (Hcy), glycine (Gly), isoleucine (Ile), alanine (Ala), phenylalanine (Phe), threonine (Thr), valine (Val), proline (Pro), and serine (Ser) were purchased from J & K Scientific Ltd. (Shanghai, China). N-methylmaleimide was purchased from Alfa Aesar. All other chemicals were of analytical grade and used without further purification. Double distilled water was used throughout the experiment.

### 2.2. Apparatus

Fluorescence spectra were collected by HITACHI F-7000 fluorescence spectrometer. Fluorescence imaging experiments were carried out by an Olympus FV1200 fluorescence microscope. NMR experiments were carried out by a Bruker AVANCE 500 NMR spectrometer.

### 2.3. Fluorescence Measurements

Fresh solution of DDQ (1 mM) was prepared prior to experiment by dissolving DDQ in ethanol; stock solution of biothiols was prepared by dissolving biothiols in double distilled water separately and further diluted to 1.0 × 10^−1^ - 1.0 × 10^−3^ M stepwise. Various 1.0 × 10^−1^ M amino acids (Gly, Ile, Ala, Phe, Thr, Val, Pro, and Ser) solutions were prepared by dissolving amino acids in double distilled water, respectively. Samples for fluorescence measurements were prepared by mixing of appropriate amount of DDQ solution and biothiols solution and then diluted to 3 ml with ethanol in 1 cm × 1 cm quartz cuvette.

### 2.4. Interaction Mechanism

In order to study the mechanism of the interaction between probe DDQ and biothiol, theoretical calculations and NMR experiments were performed. Using the density functional theory, the ground state structures of molecules were optimized and the energies were calculated at the level of PBEPBE/6-31+G (d). The solvent model used was polarizable continuum model (PCM). All the calculations were performed by Gaussian09 program package.

### 2.5. Confocal Imaging

In order to explore the presence and status of biothiols in living cells, the confocal fluorescence imaging experiment was carried out on Eca9706 cells, which were seeded on 35 mm glass-bottomed dishes for 24 h before imaging. Eca9706 cells were treated with 0.66 mM DDQ at 37°C for 40 min, then washed three times with PBS buffer solution, and imaged. Contrary to that, in the control group, Eca9706 cells were pretreated with 1 mM N-methylmaleimide (a masking reagent for biothiols) at 37°C for 30 min, then rinsed with preheated buffer for three times and incubated with 0.15 mg/ml of probe DDQ at 37°C for 40 min, washed with PBS, and imaged subsequently. The confocal fluorescence imaging experiments were carried out by an Olympus FV1200 fluorescence microscope with excitation at 405 nm. 

## 3. Results and Discussion

### 3.1. Fluorescence Response of Biothiols

**Figure 1:**
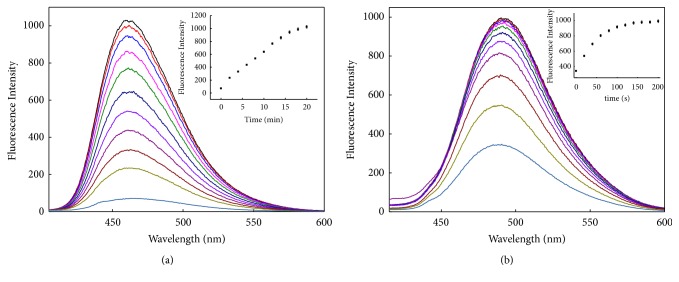
Change of fluorescence spectra with time for the reactions of DDQ and biothiols in the ethanol system. (a) DDQ (10 *μ*M) with Hcy (0.1 mM), (inset) change of fluorescence intensity with time at 467 nm. (b) DDQ (10 *μ*M) with GSH (1 mM), (inset) change of fluorescence intensity with time at 494 nm.

The fluorescence measurements were performed at excitation wavelength 390 nm and emission wavelength 405-600 nm. DDQ itself has weak fluorescence and its emission wavelength is 441 nm. Upon addition of Hcy to DDQ in the system, a dramatic rise in the fluorescence was observed, the fluorescence intensity at 467 nm enhanced with time increasing (Figure 1(a)), and the maximal fluorescence intensity reached 1026 at the 20th minute; in this system, the concentration of DDQ was 10 *μ*M and that of Hcy was 0.1 mM. Under the same excitation wavelength 390 nm, when Hcy was substituted by Cys and then reacted with DDQ, similar phenomenon occurred and its emission wavelength was also 467 nm; the maximal fluorescence intensity of 839 postponed to the 33th minute (Figure S1a).

When DDQ interacted with GSH (1 mM), the emission wavelength was located at 494 nm and the fluorescence intensity increased significantly; it was found that the interaction was very fast and the fluorescence intensity reached to the maximal value within 3 minutes (Figure 1(b)). When the concentration of GSH was raised to 2 mM, the interaction reached equilibrium in just one minute (Figure S1b).

### 3.2. Analytical Performance of DDQ

**Figure 2:**
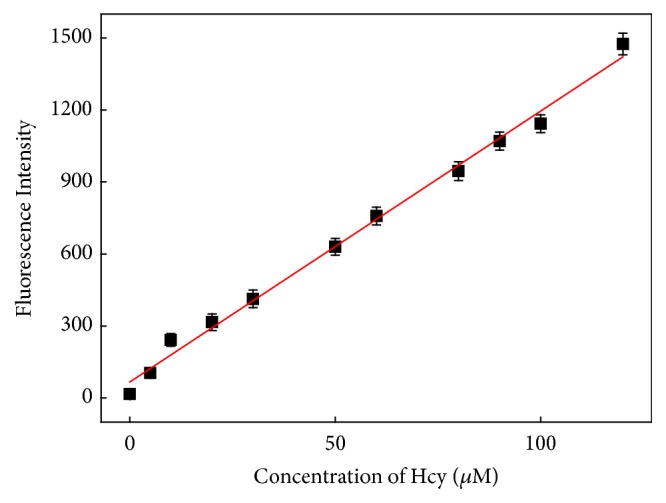
Changes of fluorescence intensity at 467 nm as the concentration increased of Hcy.

When DDQ reacted with different concentrations of GSH/Hcy/Cys, respectively, it was found that the fluorescence intensity at 467 nm is linearly related to the concentration of Hcy in the range of 0~120 *μ*M (0, 5, 10, 20, 30, 50, 60, 80, 90, 100, and 120 *μ*M) (Figure 2), with an equation F=11.29 × 10^6^C+66.62 (F represents the measured fluorescence intensity and C stands for the concentration of biothiol), *R*^2^ = 0.9930. Its detection limit is 2.17 × 10^−9^ M for Hcy, according to the formula LOD = 3S_a_/b [42], where S_a_ is the standard deviation of the response and b is the slope of the calibration curve.

Similarly, the detection limit 8.55 × 10^−8^ M for Cys and the dynamic range 0~150 *μ*M (Figure S2 a) and the detection limit 3.08 × 10^−9^ M for GSH and the dynamic range 0~60 *μ*M (Figure S2 b) were obtained.

From Table 1 it could be seen that probe DDQ has low detection limits for biothiols and responses fast to GSH and it can differentiate GSH from the other two biothiols. Structures of the probes were shown in Figure S4.

### 3.3. Selectivity of the Detection

**Figure 3:**
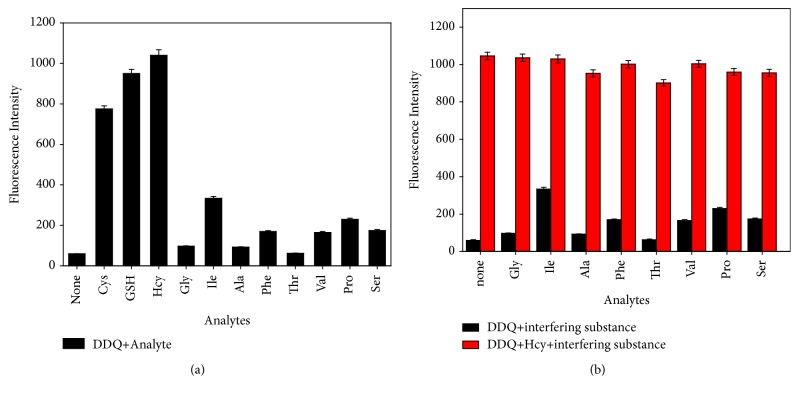
Fluorescence responses of DDQ to different substances. (a) Fluorescence responses of DDQ (10 *μ*M) to different amino acids (Cys is 0.2 mM, Hcy is 0.1 mM, and GSH and nonthiol-containing amino acids are 1 mM). (b) The black and red histogram represent the fluorescence intensity of DDQ (10 *μ*M) in the presence of various alalytes (1 mM) before and after addition of Hcy (0.1 mM).

To evaluate the selectivity of probe DDQ for biothiols, nonthiol-containing amino acids (Gly, Ile, Ala, Phe, Thr, Val, Pro, and Ser) were selected as interfering substances to assess the selectivity of DDQ. When DDQ reacted with those nonthiol-containing amino acids, only a weak fluorescence was produced (Figure 3(a)). When DDQ worked with biothiols, a strong fluorescence occurred and the fluorescence intensity was hardly affected when the interfering substance was then added to the system (Figures 3(b) and S3). Results indicated that DDQ has a good selectivity for thiol-containing compounds.

### 3.4. Response Mechanism

#### 3.4.1. Intermolecular Interaction between DDQ and Cys

**Figure 4:**
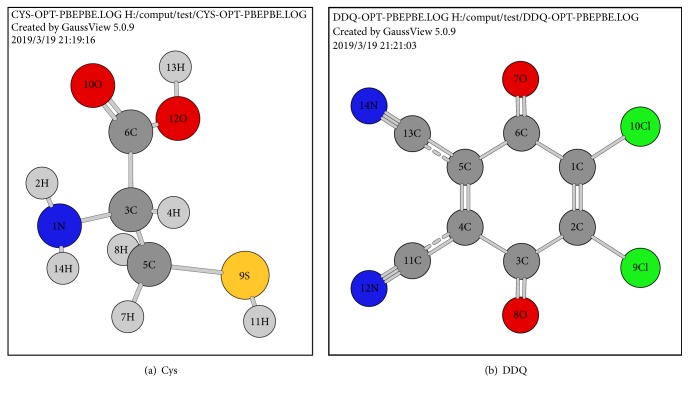
The optimized structures of Cys (a) and DDQ (b).

**Figure 5:**
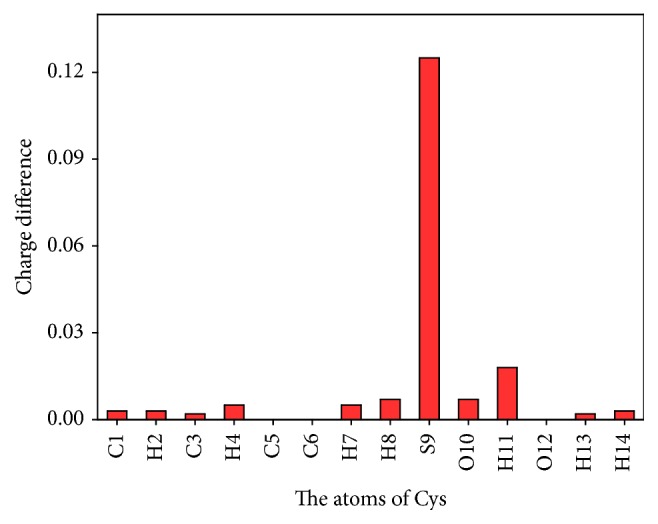
The charge difference of each atom of Cys.

In order to investigate the interaction between DDQ and Cys, the ground state structures of Cys, DDQ, and the intermolecular interaction system formed by them were optimized (Figure 4). Based on the optimized structure, the molecular natural bond orbit (NBO) charge was calculated, as shown in Table 2. In the interactive system formed by Cys and DDQ, Cys had a positive charge of +0.180 and DDQ had a negative charge of -0.180, indicating that there was a significant electrostatic interaction between Cys and DDQ. The single point energy calculation proved that the interaction forces between Cys and DDQ were 15.57 kJ/mol, which was consistent with the range of electrostatic attraction in intermolecular force (12-20 kJ/mol), so the type of forces between Cys and DDQ was electrostatic interaction. From the change of atomic charge of Cys before and after the interaction, it could be seen that the charge of S9 increased from -0.095 to 0.030, the increment was 0.125 and S9 was the source of electron donor, and the electrons were transferred from S9 of Cys to DDQ (Figure 5). The transferred electrons did not concentrate on a single atom in DDQ because of the electronic delocalization dispersion, making the atomic charge of C5, C4, C3, N12, N14, O7, and O8 and other atoms had a certain degree of reduction. In DDQ, it contained two electron-withdrawing groups of -Cl and -CN; in that case the electron density of the conjugate part was low and the fluorescence was weak. -SH is an electron rich group, when the two compounds interacted with each other; the electrons transferred from -SH of Cys to DDQ, thus resulting in the increase of the electron cloud density and significant enhancement of fluorescence intensity.

#### 3.4.2. Molecular Orbital Analysis

**Figure 6:**
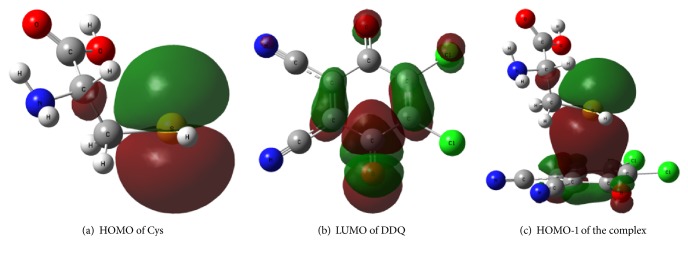
Molecular frontier orbital diagrams of Cys, DDQ, and the complex.

To further confirm that the interaction force between biothiol and DDQ is intermolecular electrostatic interaction, the frontier molecular orbitals of Cys, DDQ, and the interaction system were obtained by single point energy calculation, as shown in Figure 6. The main contribution of the highest occupied molecular orbital (HOMO) of Cys came from the p orbit of S9 (Figure 6(a)), while the lowest unoccupied molecular orbital (LUMO) of DDQ was the conjugate *π∗* orbit (Figure 6(b)). From the HOMO-1 of the interaction system formed by Cys and DDQ, it could be seen that there was a certain degree of overlap between the Cys orbit and the DDQ orbit (Figure 6(c)), which confirmed that the interaction force between two molecules was electrostatic interaction [43].

#### 3.4.3. H-NMR Experiment

**Figure 7:**
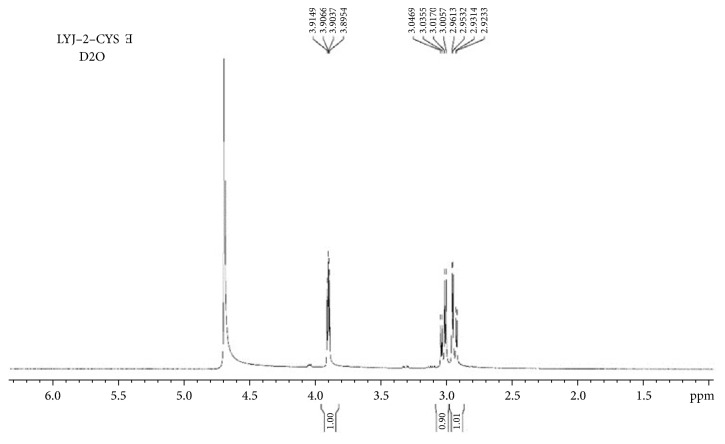
H-NMR spectra of Cys in D_2_O.

**Figure 8:**
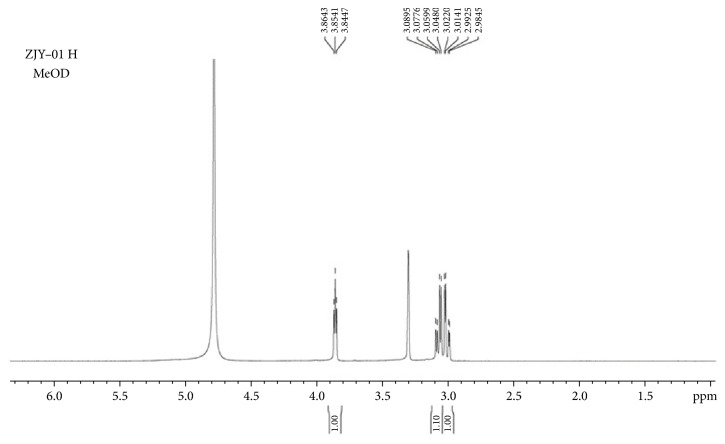
H-NMR spectra of Cys and DDQ in MeOD.

H-NMR experiments were performed to verify the interaction mechanism of Cys and DDQ (Figures 7 and 8). In Figure 7 the chemical shift of H7 and H8 is 3.03 and 2.95 ppm; after the interaction with DDQ, the chemical shift of H7 and H8 moved slightly to a lower field (3.07 and 3.01 ppm), which indicated that the electron cloud density around hydrogen nucleus decreased and resonance peak moved to the low field. This was consistent with the results of theoretical calculations, -SH is an electron rich group, and S9 was the source of electron donor, when Cys interacted with DDQ; the electrons were transferred from S9 of Cys to DDQ, resulting in the increase of the electron cloud density and significant enhancement of fluorescence intensity.

DDQ takes p-benzoquinone as the basic structure. When there is no substituent in the p-benzoquinone structure, it can take place nucleophilic reaction with biothiol. In the structure of DDQ, substituents -Cl, -Cl, -CN, and -CN are attached to 1C, 2C, 4C, and 5C, respectively, which makes it difficult to undergo nucleophilic substitution reaction. When the interaction between DDQ and biothiol is insufficient to break the old chemical bond, the substitution reaction will not occur. H-NMR spectra showed that, except for the slight change of chemical shifts of H7 and H8, no new chemical shifts of H appeared. Therefore, we conclude that no new compound was produced after the interaction between Cys and DDQ, and the interaction force between them is electrostatic attraction.

### 3.5. MTT Assay

The cytotoxicity of DDQ was evaluated by MTT assay. The human esophageal cancer Eca9706 cells were seeded at a density of 4 × 10^4^ cells/mL in 96-well microplates (200 *μ*L/well) and cultured for 24 h. The cells were treated with the probe solution for 48 h. Subsequently, 20 *μ*L portion of MTT (5 mg/mL) in PBS buffer was added to each well and incubated for another 4 h. The solution was removed and the cells were washed with PBS. The DMSO (150 *μ*L/well) was added with shaking for 10 min. The absorbance was measured at 570 nm using a microplate reader. The cytotoxicity of DDQ was measured as the percentage ratio of the absorbance of the treated cells over the untreated controls. The IC_50_ values were calculated by SPSS statistical software; IC_50_ is 51.51±0.21 *μ*g/ml.

### 3.6. Confocal Imaging

In order to verify the potential application of DDQ for biothiols imaging in living cells, Eca9706 cells were incubated with probe DDQ for 40 min and observed by confocal fluorescence microscope.

**Figure 9:**
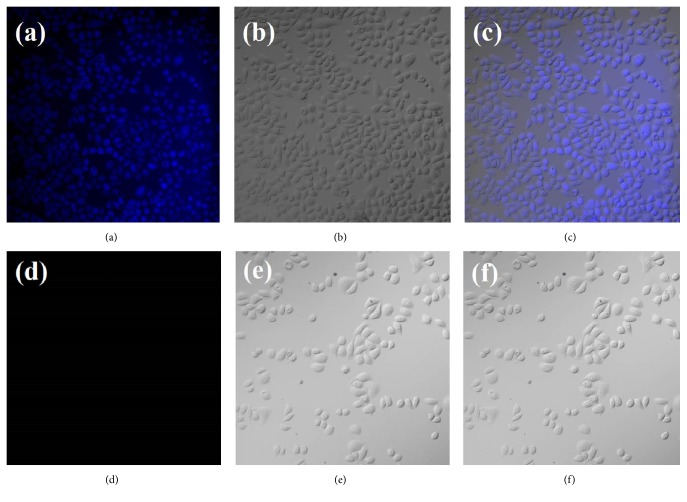
Confocal laser scanning microscopy images of living Eca9706 cells. (a) Fluorescence image of Eca9706 cells incubated with 0.15 mg/ml DDQ for 40 min at 37°C. (b) Bright-field transmission image of Eca9706 cells shown in panel (1). (c) Overlay image of (a) and (b). (d) Fluorescence image of Eca9706 cells pretreated with 1mM N-methylmaleimide (a masking reagent for thiols) at 37°C for 30 min and then cultured with 0.15 mg/ml DDQ for 40 min at 37°C. (e) Bright-field transmission image of Eca9706 cells shown in panel (d). (f) Overlay image of (d) and (e).

As shown in Figure 9(a), a bright blue fluorescence was observed inside the cells, indicating that the probe DDQ is capable of permeating into cells and reacting with biothiols in Eca9706 cells to generate fluorescence.  Figure 9(c) is the overlay image of fluorescence image Figure 9(a) and bright-field transmission image Figure 9(b). In contrast, the Eca9706 cells in the control group were pretreated with 1 mM N-methylmaleimide (a well-known masking reagent for biothiols) before an incubation with DDQ; there was no fluorescence emitted (Figures 9(d)–9(f)); this demonstrated that DDQ has good selectivity for biothiols; it did not react with other substances in the cells; and thus the specific detection of biothiols was obtained. These results suggest that DDQ can serve as a promising fluorescent probe for biothiols imaging in living cells.

## 4. Conclusions

In summary, the new fluorescent probe DDQ exhibits excellent properties such as good sensitivity, high selectivity, and low detection limits for the detection of biothiols. It can distinguish glutathione from the other two biothiols and can detect glutathione within three minutes. The interaction forces between the probe DDQ and biothiols are electrostatic interaction; it makes the electron cloud density of DDQ increase; thus the fluorescence intensity is significantly enhanced. More importantly, it has been successfully applied to the confocal imaging of biothiols in Eca9706 cells, suggesting that it has a promising prospect for diagnostic-related application.

## Figures and Tables

**Table 1:** Comparison of DDQ to other fluorescent probes for biothiols detection.

Probe	*λ*ex	*λ*em	Detection limit	Response time	Reference
A	360 nm	423 nm	8.4 × 10^−8^ M for Cys 8.0 × 10^−9^ M for GSH	30 min for Cys 15 min for GSH	[17]

B	400 nm	530 nm	5.0 × 10^−7^ M for Cys	50 min for Cys	[18]

CyR	698 nm	735 nm	1.50 × 10^−7^ M for GSH	within 180 s for GSH	[19]

DMDP-M	385 nm	470 nm	1.80 × 10^−7^ M for Cys	10 min for Cys	[20]

C	438 nm for Cys, 537 nm for GSH	515 nm for Cys 567 nm for GSH	1.1 × 10^−7^ M for Cys; 5.0 × 10^−9^ M for GSH	10 min for Cys 120 s for GSH	[21]

D	450 nm	545 nm	3.5 × 10^−8^ M for Hcy 5.3 × 10^−8^ M for Cys 5.2 × 10^−8^ M for GSH	20 s for Hcy, Cys 5 min for GSH	[22]

DDQ	390 nm	467 nm for Cys and Hcy; 494 nm for GSH	2.17 × 10^−9^ M for Hcy 8.55 × 10^−8^ M for Cys 3.08 × 10^−9^ M for GSH	33 min for Cys 20 min for Hcy within 180 s for GSH	This work

**Table 2:** Distribution of atomic NBO charge before and after the interaction of Cys and DDQ.

The atom of Cys	The charge of atom of Cys before interaction	The charge of atom of Cys after interaction	Difference	The atom of DDQ	The charge of atom of DDQ before interaction	The charge of atom of DDQ after interaction	Difference
N1	-0.935	-0.932	0.003	C1	-0.123	-0.124	-0.001
H2	0.422	0.425	0.003	C2	-0.123	-0.122	0.001
C3	-0.210	-0.208	0.002	C3	0.488	0.475	-0.013
H4	0.275	0.280	0.005	C4	-0.106	-0.118	-0.012
C5	-0.630	-0.630	0.000	C5	-0.106	-0.140	-0.034
C6	0.774	0.774	0.000	C6	0.488	0.478	-0.010
H7	0.287	0.292	0.005	O7	-0.448	-0.472	-0.024
H8	0.288	0.295	0.007	O8	-0.448	-0.472	-0.024
S9	-0.095	0.030	0.125	Cl9	0.149	0.139	-0.010
O10	-0.613	-0.606	0.007	Cl10	0.149	0.134	-0.015
H11	0.174	0.192	0.018	C11	0.279	0.281	0.002
O12	-0.685	-0.685	0.000	N12	-0.239	-0.257	-0.018
H13	0.532	0.534	0.002	C13	0.279	0.283	0.004
H14	0.416	0.419	0.003	N14	-0.239	-0.265	-0.026
Total	0.000	0.180	0.180		0.000	-0.180	-0.180

## Data Availability

The data used to support the findings of this study are included within the article and supplementary information file.

## Supplementary Materials

Supplementary Materials
* Figure S1*: Change of fluorescence spectra with time for the reactions of DDQ and biothiols in the ethanol system. (a) DDQ (10 *μ*M) with Cys (0.2 mM), (inset) change of fluorescence intensity with time at 467 nm. (b) DDQ (10 *μ*M) with GSH (2 mM), (inset) change of fluorescence intensity with time at 494 nm.* Figure S2*: Change of fluorescence intensity with concentration of biothiol. (a) Cys. (b) GSH.* Figure S3*: fluorescence responses of DDQ to different substances. The black histogram represents the fluorescence intensity of the reaction between DDQ (10 *μ*M) and interfering substance (1 mM). The red histogram represents the fluorescence intensity after adding the interfering substance to the reaction between DDQ and thiol. (a) Cys (0.2 mM). (b).* Figure S4*: structures of the probes in Table 1.
