# Supplementary material for: A New Quinone Based Fluorescent Probe for High Sensitive and Selective Detection of Biothiols and Its Application in Living Cell Imaging
Source: Int J Anal Chem. 2019 Apr 9;2019:7536431. doi: 10.1155/2019/7536431 (PMC6481154; doi:10.1155/2019/7536431)
Supplement: Supplementary Materials — Figure S1: Change of fluorescence spectra with time for the reactions of DDQ and biothiols in the ethanol system. (a) DDQ (10 μM) with Cys (0.2 mM), (inset) change of fluorescence intensity with time at 467 nm. (b) DDQ (10 μM) with GSH (2 mM), (inset) change of fluorescence intensity with time at 494 nm. Figure S2: Change of fluorescence intensity with concentration of biothiol. (a) Cys. (b) GSH. Figure S3: fluorescence responses of DDQ to different substances. The black histogram represents the fluorescence intensity of the reaction between DDQ (10 μM) and interfering substance (1 mM). The red histogram represents the fluorescence intensity after adding the interfering substance to the reaction between DDQ and thiol. (a) Cys (0.2 mM). (b). Figure S4: structures of the probes in Table 1. [file 7536431.f1.docx]

**A new quinone based fluorescent probe for high sensitive**

**and selective detection of biothiols and its application in living cell imaging**

Yanju Liu^#1^, Manman Li^#1^, Keith Man-Chung Wong^3^, Yan Tong^1^,

Huaixia Yang***^1^, Jinming Kong***^2^

^1^ Pharmacy College, Henan University of Chinese Medicine, Zhengzhou 450008, P. R. China.

^2^ School of Environmental and Biological Engineering, Nanjing University of Science and

Technology, Nanjing 210094, P. R. China.

^3^ Department of Chemistry, Southern University of Science and Technology, Shenzhen 518055, P.R. China.

* Corresponding authors

Huaixia Yang, E-mail: yhx998@hactcm.edu.cn

Jinming Kong, E-mail: j.kong@njust.edu.cn. Tel.: +86-25-84303109, Fax: +86-25-84303109

^#^ Yanju Liu and Manman Li contributed equally to this study and share first authorship.

1. **Cover letter**

Dear Editor,

We would like to submit the enclosed manuscript entitled “**A new quinone based fluorescent probe for high sensitive and selective detection of biothiols and its application in living cell imaging**”, which we wish to be considered for publication in **International Journal of** Analytical Chemistry as a research paper.

Thiol-containing biomolecules like cysteine (Cys), glutathione (GSH) and homocysteine (Hcy) take participate in a variety of physiological processes, are essential substances in biological systems, and the connection of generation and metabolism pathways among them is very closely. Discriminating biothiols from each other is still be a challenge, which mainly because of the similar chemical structures and properties of them.

The fluorescence method has attracted much attention due to its great temporal and spatial sampling capability as well as high sensitivity. Herein, we present a novel fluorescent probe 2,3-dichloro-5,6-dicyano-1,4-benzoquinone (DDQ). Compared with similar probes, this probe has fast response to three kinds of biothiols and can differentiate glutathione through emission wavelength. The detection of biothiols has not been affected in the presence of some other amino acids, indicating that it has good selectivity for biothiols. The detection limit was determined to be 3.08×10^-9^ M for glutathione, 8.55×10^-8^ M for cysteine, 2.17×10^-9^ M homocysteine, respectively.

We further explored the sensing mechanism with density functional theory, and the results demonstrated that the interaction forces between the probe and biothiols were electrostatic forces. In addition, DDQ was also successfully applied to detection of biothiols in Eca9706 cells by fluorescence imaging.

We are confident that our manuscript fits the criteria of **International Journal of** Analytical Chemistry**.** In addition, the manuscript has been prepared in accordance with the instruction of **International Journal of** Analytical Chemistry. The work described has not been submitted elsewhere for publication, in whole or in part, and all the authors listed have approved the manuscript that is enclosed. We have read and have abided by the statement of ethical standards for manuscripts submitted to **International Journal of** Analytical Chemistry.

Thank you very much for your attention and consideration. We look forward to receiving your acknowledgment and hearing the outcome of the reviews from you. If you have any queries, please don’t hesitate to contact me at the address below.

With our kindest regards!

Yours sincerely,

**Prof. Huaixia Yang**

Pharmacy College,

Henan University of Chinese Medicine,

Zhengzhou 450008, P. R. China.

E-mail: yhx998@hactcm.edu.cn

**Prof. Jinming Kong**

School of Environmental and Biological Engineering

Nanjing University of Science and Technology

Nanjing 210094, P. R. China

E-mail: j.kong@njust.edu.cn

Tel: +86-25-84303109

Fax: +86-25-84303109

2. **Supporting information**

|  |  |
| --- | --- |
| **Fig. S1** Time-dependent fluorescence intensity change of DDQ and biothiol. (a) DDQ (10 μM) and Cys（0.2 mM）at 467 nm, (inset) change of fluorescence spectra. (b) DDQ (10 μM) and GSH（2mM）at 494 nm, (inset) change of fluorescence spectra. | |

|  |  |
| --- | --- |
| **Fig. S2** Changes of fluorescence intensity with concentration of biothiol. (a) Cys. (b) GSH. | |

|  |  |
| --- | --- |
| **Fig. S3** Fluorescence responses of DDQ to different substances. The black histogram represents the fluorescence intensity of the reaction between DDQ (10 μM) and interfering substance (1 mM). The red histogram represents the fluorescence intensity after adding the interfering substance to the reaction between DDQ and thiol. (a) Cys (0.2 mM). (b) GSH (1 mM). | |

|  |  |
| --- | --- |
| Probe A | Probe B |
|  |  |
| Probe C | Probe D |
|  |  |
| Probe CyR | Probe DMDP-M |
| **Fig. S4** Structures of the probes in Table 1. | |
